# Fluoromycobacteriophages Can Detect Viable *Mycobacterium tuberculosis* and Determine Phenotypic Rifampicin Resistance in 3–5 Days From Sputum Collection

**DOI:** 10.3389/fmicb.2018.01471

**Published:** 2018-07-05

**Authors:** Liliana Rondón, Estefanía Urdániz, Cecilia Latini, Florencia Payaslian, Mario Matteo, Ezequiel J. Sosa, Darío F. Do Porto, Adrian G. Turjanski, Sergio Nemirovsky, Graham F. Hatfull, Susana Poggi, Mariana Piuri

**Affiliations:** ^1^Departamento de Química Biológica, Facultad de Ciencias Exactas y Naturales, Universidad de Buenos Aires, Instituto de Química Biológica de la Facultad de Ciencias Exactas y Naturales – Consejo Nacional de Investigaciones Científicas y Técnicas, Buenos Aires, Argentina; ^2^Instituto de Tisioneumonología Raúl F. Vaccarezza, Hospital de Infecciosas Dr. F. J. Muñiz, Buenos Aires, Argentina; ^3^Plataforma de Bioinformática Argentina, Instituto de Cálculo, Facultad de Ciencias Exactas y Naturales, Universidad de Buenos Aires, Buenos Aires, Argentina; ^4^Department of Biological Sciences and Pittsburgh Bacteriophage Institute, University of Pittsburgh, Pittsburgh, PA, United States

**Keywords:** tuberculosis, diagnosis, fluoromycobacteriophages, drug susceptibility testing, *mCherry_bomb_*Φ

## Abstract

The World Health Organization (WHO) estimates that 40% of tuberculosis (TB) cases are not diagnosed and treated correctly. Even though there are several diagnostic tests available in the market, rapid, easy, inexpensive detection, and drug susceptibility testing (DST) of *Mycobacterium tuberculosis* is still of critical importance specially in low and middle-income countries with high incidence of the disease. In this work, we have developed a microscopy-based methodology using the reporter mycobacteriophage *mCherry_bomb_*ϕ for detection of *Mycobacterium spp.* and phenotypic determination of rifampicin resistance within just days from sputum sample collection. Fluoromycobacteriophage methodology is compatible with regularly used protocols in clinical laboratories for TB diagnosis and paraformaldehyde fixation after infection reduces biohazard risks with sample analysis by fluorescence microscopy. We have also set up conditions for discrimination between *M. tuberculosis* complex (MTBC) and non-tuberculous mycobacteria (NTM) strains by addition of *p*-nitrobenzoic acid (PNB) during the assay. Using clinical isolates of pre-XDR and XDR-TB strains from this study, we tested *mCherry_bomb_*Φ for extended DST and we compared the antibiotic resistance profile with those predicted by whole genome sequencing. Our results emphasize the utility of a phenotypic test for *M. tuberculosis* extended DST. The many attributes of *mCherry_bomb_*Φ suggests this could be a useful component of clinical microbiological laboratories for TB diagnosis and since only viable cells are detected this could be a useful tool for monitoring patient response to treatment.

## Introduction

There are substantial advances in diagnosis and treatment of tuberculosis (TB), but this disease remains a significant global health threat (Sulis et al., 2016). The World Health Organization (WHO) estimates that 40% of TB cases go undiagnosed and consequently not treated (Glaziou et al., 2013; López-Hernández et al., 2016). In 2016, WHO recommended the use of rapid molecular tests to speed up TB drug susceptibility testing (DST) (WHO, 2016) although due to the cost of equipment and supplies, Ziehl Neelsen staining of *Mycobacterium spp.* in sputum, with subsequent culture to determine viable bacilli and DST using the proportion method is often the method of choice. Culture methodology is laborious and takes 3–6 weeks to report the presence of viable mycobacteria in the sample and a few additional weeks for DST (Naveen and Peerapur, 2012).

Mycobacteriophages are viruses that efficiently and specifically infect *Mycobacterium spp.*, and provide tools for genetic analysis and clinical control of TB (Hatfull, 2010, 2012). Several phage-based diagnostic approaches have been described, including the commercially developed phage amplification biological assay (FASTPlaque^TM^) utilizing *M. tuberculosis*-dependent reproduction of phage D29 and plaque formation on the fast-growing *Mycobacterium smegmatis* (Wilson et al., 1997; Watterson et al., 1998). The FASTPlaque assay has been adapted for determining resistance to rifampicin and other antibiotics commonly used for TB treatment (Albert et al., 2001).

An alternative luciferase reporter phage assay uses recombinant mycobacteriophages carrying the firefly luciferase gene to detect *M. tuberculosis* by luminescence, coupled with empirical determination of drug resistance by light emission in the presence of antibiotic (Jacobs et al., 1993). These phages provide a simple and rapid assay, but are not well-suited to detection of partially resistant cultures; they also require the propagation of live potentially infectious cultures, and the level of sensitivity in laboratory grown cultures has yet to be fully replicated with clinical specimens (Eltringham et al., 1999; Banaiee et al., 2001; Dinnes et al., 2007; WHO, 2017).

Fluoromycobacteriophages are reporter phages containing a fluorescent reporter gene such as *gfp* or *ZsYellow* (Piuri et al., 2009) and has potential for rapid diagnosis and DST of TB clinical isolates (Rondón et al., 2011). These phages are thermosensitive and do not lyse cells at 37°C, and in consequence, fluorescent mycobacterial cells can be readily detected by fluorescence microscopy or flow cytometry. Recently, we described the construction of a second generation fluoromycobacteriophage, *mCherry_bomb_*ϕ, with optimized expression of a *mCherry_bomb_* gene in mycobacteria with improved fluorescent signal and shorter time to detection of *M. tuberculosis* using fluorimetry (Urdániz et al., 2016).

The current fluoromycobacteriophages are derived from phage TM4, a broad host range phage that can infect several Mycobacterial species including, *M. tuberculosis*, *M. bovis*, *M. smegmatis*, and atypical Mycobacteria (Ford et al., 1998). They can rapidly and easily reveal the metabolic state of *M. tuberculosis* and consequently report its response to antibiotics (Piuri et al., 2009; Rondón et al., 2011), and have potential advantages over existing phage-based TB diagnostic methods. Notably, after reporter phage infection, cells can be fixed using paraformaldehyde allowing safe manipulation of the sample and preservation of the fluorescence (Piuri et al., 2009; Jain et al., 2012; Yu et al., 2016). Fluorescence microscopy provides for simple sample analyses, and low-cost LED (light emitting diode) fluorescence adapters facilitate the use of fluorescence microscopy in developing countries (Anthony et al., 2006; Marais et al., 2008). Moreover, Breslauer et al. (2009) described the use of a mobile phone-mounted fluorescence microscope with LED excitation with potential for imaging *M. tuberculosis*-infected samples.

Next generation sequencing and our understanding of the genetic basis of antibiotic resistance has led to the development of rapid genotypic TB tests (Cole et al., 1998; Brossier et al., 2017). These methods can offer high sensitivity, even for paucibacillary samples, and do not require culture (Garberi et al., 2011; Teo et al., 2011; Mboowa et al., 2014; Rufai et al., 2014; Faksri et al., 2015; Sales et al., 2015; Rahman et al., 2016). Xpert and MTBDR-plus allow detection of *M. tuberculosis* and determination of rifampicin resistance (RIF^R^) directly in sputum samples within hours, and is recommended by the World Health Organization (WHO, 2011; Ocheretina et al., 2014; Rahman et al., 2016). However, this technology may still be cost prohibitive in countries with moderate to high incidence of TB, limiting its application to samples from patients with risk factors associated to the disease or presumptively carrying *M. tuberculosis* resistant strains (Blakemore et al., 2010; Teo et al., 2011; WHO, 2011; Chang et al., 2012; Rufai et al., 2014; Rahman et al., 2016). In addition, some discrepancies remain between genotypic and phenotypic tests (Ocheretina et al., 2014; Mokaddas et al., 2015; Gurbanova et al., 2017), and at least in some clinical isolates, resistance cannot be explained by the presence of known gene mutations (Hazbón, 2004; Louw et al., 2009).

Here, we report evaluation of the new *mCherry_bomb_*ϕ for detection of *M. tuberculosis* and rifampicin resistance in sputum samples from presumptive TB patients in Buenos Aires city. Also, using fluorescence microscopy for detection, we evaluated the use of *p*-nitrobenzoic acid (PNB) for selective inhibition of members of the *M. tuberculosis* complex (MTBC) (Sharma et al., 2010; Shakoor et al., 2010) to distinguish MTBC from atypical or non-tuberculous mycobacteria (NTM). Additionally, we established conditions for determination of susceptibility to other antibiotics that discriminate between multi-drug resistant TB (MDR-TB), strains wich are resistant to rifampicin and isoniazid; extremely drug resistant TB (XDR- TB), resistant to rifampicin, isoniazid, plus any fluoroquinolone and an injectable drug, kanamycin, amikacin or capreomycin, or pre-XDR wich show resistance to rifampicin, isoniazid and a fluoroquinolone or an injectable drug. Whole genome sequencing (WGS) of XDR-TB strains identified in this study allowed comparison between phenotypic and genotypic results, and underscore the importance of rapid methods that evaluate phenotypic resistance in clinical isolates.

## Materials and Methods

### Study Population

Two hundred and eighty-three adult presumptive TB patients, treatment-naive, under antibiotic treatment or that were previously treated admitted at Laboratorio de Bacteriología de Tuberculosis, Instituto de Tisioneumonología Dr. Raul Vaccarezza, Hospital Muñiz, Ciudad Autónoma de Buenos Aires, Argentina were included in the study. All the participants signed written informed consent forms, previously approved by the Ethics Committee of Instituto Vaccarezza, to participate. The study included adult patients of different nationalities (mostly Argentinean and from neighboring countries) and gender, with different classifications at the time of sample taking: treatment-naive, under treatment, discontinued treatment, relapsed, treatment failure, etc. Only pulmonary samples were analyzed.

### Sputum Samples

Sputum samples were collected in a sterile container and decontaminated using a modified Petroff protocol. Briefly, after addition of one volume of 4% NaOH the sample was incubated at room temperature for 15 min. After incubation, it was centrifuged for 20 min, the supernatant was discarded, and the pellet was neutralized to pH 7 by dropwise addition of 15% H_2_SO_4_. The pellet was washed and resuspended with distilled water. The pellet was used for routine tests performed in Laboratorio de Bacteriología de la Tuberculosis, including Ziehl Neelsen stain (Weldu et al., 2013) inoculation on Löwenstein Jensen (LJ) media for detection and colony counting of mycobacteria and inoculation on Stonebrink media (LJ supplemented with 2% sodium pyruvate) for detection of *M. bovis* (Daza Bolaños et al., 2017). Only for samples of patients at high-risk of developing TB disease an aliquot was used for analysis with MGIT 960 (Martin et al., 2009). Finally, the remnant of the pellet was used to test with *mCherry_bomb_*ϕ.

### Preparation of Stocks of *mCherry_bomb_*ϕ

In this study, we used the second generation fluoromycobacteriophages, carrying an *mCherry_bomb_* gene with optimized expression in mycobacteria (Urdániz et al., 2016). Stocks of *mCherry_bomb_*ϕ were prepared as previously described (Rondón et al., 2011).

### Evaluation of *mCherry_bomb_*ϕ for Detection and Determination of RIF Susceptibility of *Mycobacterium spp.*

Remnant pellets of processed sputum samples were resuspended in 400 μl of Middlebrook 7H9 broth supplemented with OADC Enrichment (Becton, Dickinson and Company, Sparks, MD, United States) and PANTA (Becton, Dickinson and Company, Sparks, MD, United States) and divided in four 100 μl aliquots. In two aliquots, 4 μg/ml RIF was added and the other two were kept as control. Samples were recovered for 48 or 96 h and infected overnight with 5 μl of a stock of *mCherry_bomb_*ϕ (10^10^–10^11^ PFU/ml). After that, samples were fixed with 2% paraformaldehyde for 2 h at room temperature, centrifuged and the pellet was resuspended in 10 μl of phosphate buffered saline (PBS) and examined by fluorescence microscopy using 1,000× magnification. The criterion for detection was the presence of at least one fluorescent bacillus per 100 fields in the control samples and for determination of RIF resistance was the same in RIF treated samples. We also compared the results using the criterion of two fluorescent bacilli per 100 field but we did not observed significantly differences between both criteria so we opted for the first one. Detection results using *mCherry_bomb_*ϕ were compared with growth and colony counting (CFU) in LJ media and determination of RIF susceptibility with the proportion method (Canetti et al., 1963).

### Microscopy and Settings

A fluorescence microscope (Axiostar Plus; Carl Zeiss) with a 100× objective with oil immersion and phase contrast was used. Fluorescence images were acquired using a Cannon EOS Rebel T3 and the software provided with the camera. For detection of fluorescent mCherry_*bomb*_ the 64 HE mPlum shift free (E) filter (EX BP 587/25, BS FT605, EM BP 647/70) (Carl Zeiss) was used. When necessary, image processing was performed using Adobe Photoshop CS2 (Adobe Systems Incorporated), but maintaining identical brightness and contrast settings for all images.

### Evaluation of PNB to Discriminate Between Mycobacteria of the MTBC and NTM

Clinical isolates from sputum samples positive for *Mycobacterium spp.* and further identified as NTM were inoculated in Middlebrook 7H9 broth supplemented with OADC and grown to exponential phase. At least two clinical isolates identified as *M. tuberculosis* were also used as controls. Two culture aliquots of 100 μl were incubated with 500 μg/ml of PNB for 24 and 48 h and one aliquot was kept as control. After incubation, samples were centrifuged and the pellet was resuspended in the same media. Samples were infected overnight at 37°C with *mCherry_bomb_*ϕ at an approximate multiplicity of infection (MOI) of 100. Samples were processed as described above and observed by fluorescence microscopy.

### Extended Antibiotic Susceptibility Testing of Pre-XDR and XDR-TB Clinical Isolates Using *mCherry_bomb_*ϕ

Four *M. tuberculosis* strains isolated from sputum samples used in this study, that tested positive for *Mycobacterium* and were RIF^R^ with *mCherry_bomb_*ϕ, were further identified as pre-XDR and XDR-TB using the proportion method. To determine the antibiotic resistance profile of these strains using our fluoromycobacteriophage methodology, 200 μl of an exponentially growing culture were preincubated for 24 h with the following antibiotics: isoniazid (0.2 μg/ml), rifampicin (4 μg/ml), streptomycin (2 μg/ml), ofloxacin (10 μg/ml), ethambutol (5 μg/ml), amikacin (1 μg/ml), kanamycin (2.5 μg/ml) and further infected overnight with *mCherry_bomb_*ϕ at an approximate MOI of 100. After incubation, samples were processed as described above and observed by fluorescence microscopy.

### DNA Isolation of Pre-XDR and XDR-TB Strains

Colonies from LJ media were used for DNA extraction. The protocol for isolation of DNA is described in van Soolingen et al. (1991). Prior to sequencing the integrity of the DNA was evaluated by gel electrophoresis (van Soolingen et al., 1991).

### Bioinformatics Analysis of Sequenced Strains

Paired end reads from the four strains analyzed were obtained using a whole-genome shotgun strategy with a MiSeq, Illumina at the Instituto Malbrán, Buenos Aires, Argentina. Sequence reads were checked for quality using FastQC version v0.11.5 (Andrews, 2011) and processed with the PRINSEQ lite version 0.20.4 (Schmieder and Edwards, 2011) for sequence read quality assessment.

All the reads were deposited in GenBank NCBI Bioproject PRJNA438689 with the following accession numbers: 152748 (ID: 8724044, 8724043), 161109 (ID: 8724038, 8724037), 156324 (ID: 8724040, 8724039), and 16138 (ID: 8724042, 8724041).

Subsequently reads were aligned to the H37Rv reference genome (NCBI access number NC_000962.3) using BWA (Li and Durbin, 2010) and variant calling was made with GATK (Mckenna et al., 2010). Resulting variants were annotated with SnpEff (Cingolani et al., 2012) and compared with previously reported drug resistance-associated variants listed in TBDREAM database (Sandgren et al., 2009). Each match was classified using the following criteria: (I) Position match, I(a): Exact match: the variant in the strain is exactly the same that the reported in TBDREAM. I(b) Partial match: the variant in the strain is in the same position that the reported in TBDREAM but present a different nucleotide. (II) Gene match: the strain has a variant in a gene reported in TBDREAM, but it is in different position. (III) No match. Analysis of the genes containing variants was performed with TuberQ database (Radusky et al., 2014). When we were not able to find a gene variant in TBDREAM that could explain the antibiotic resistant phenotype, we manually inspected other genes that have been previously reported in resistant isolates.

On the other hand, a short phylogenetic analysis was performed between the samples mentioned above and samples that were previously reported as M, Ra and Beijing sublineages downloaded from NCBI SRA: ERR760768, ERR776666, and SRR5314268, respectively. Isolates reads were processed using the variant calling pipeline mentioned above. At last, resulting variants from all samples were combined in a single VCF using GATK and a dendrogram was built using VCFKit (Cook and Andersen, 2017) with UPGMA algorithm using Beijing sample as outgroup. **Figure 4** was built using ETE Toolkit (Huerta-cepas et al., 2016).

### Statistical Analysis

Data analysis for calculation of sensitivity, specificity, and predictive values was performed using the R language (The R Foundation, 2018), and the exact 95% confidence intervals were estimated using the Hmisc library. To compare the performance of *mCherry_bomb_*ϕ and culture growth in LJ media for detection of *Mycobacterium spp.* we employed box and whisker plots.

To evaluate the correlation of *mCherry_bomb_*ϕ based detection and *Mycobacterium spp.* growth in LJ we applied generalized linear models using the negative binomial distribution. When needed multiple comparisons were performed by fitting a negative binomial models to each pair of categories of growth in LJ media using the MASS (Venables and Ripley, 2002) library for R, and adjusting the *p*-values with the Bonferroni correction. χ^2^ goodness of fit tests showed data corresponded to the negative binomial (in contrast, when a Poisson model was used this test showed a poor fitting). When needed, incidence rate ratios (IRRs) were calculated as the exponential of the model’s coefficient.

## Results

### Detection of *Mycobacterium spp.* in Sputum Samples With *mCherry_bomb_*ϕ

**FIGURE 1:**
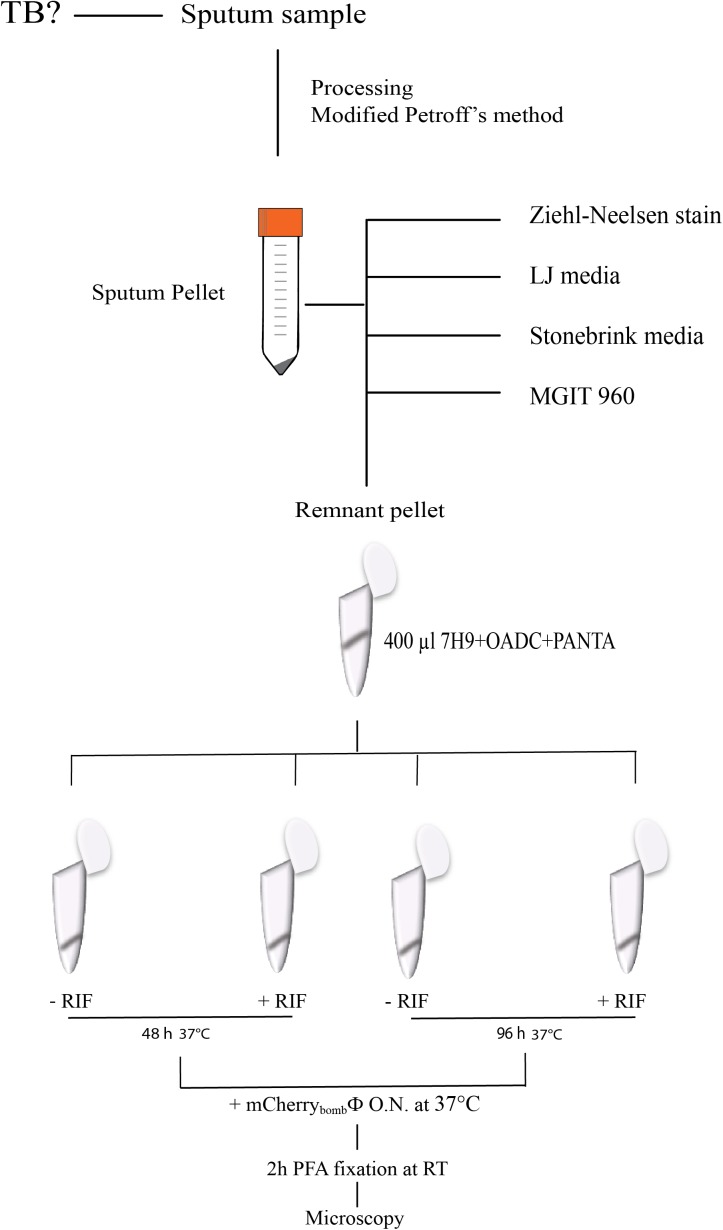
Schematic representation of the protocol for infection with *mCherry_bomb_*ϕ in processed sputum samples.

**FIGURE 2:**
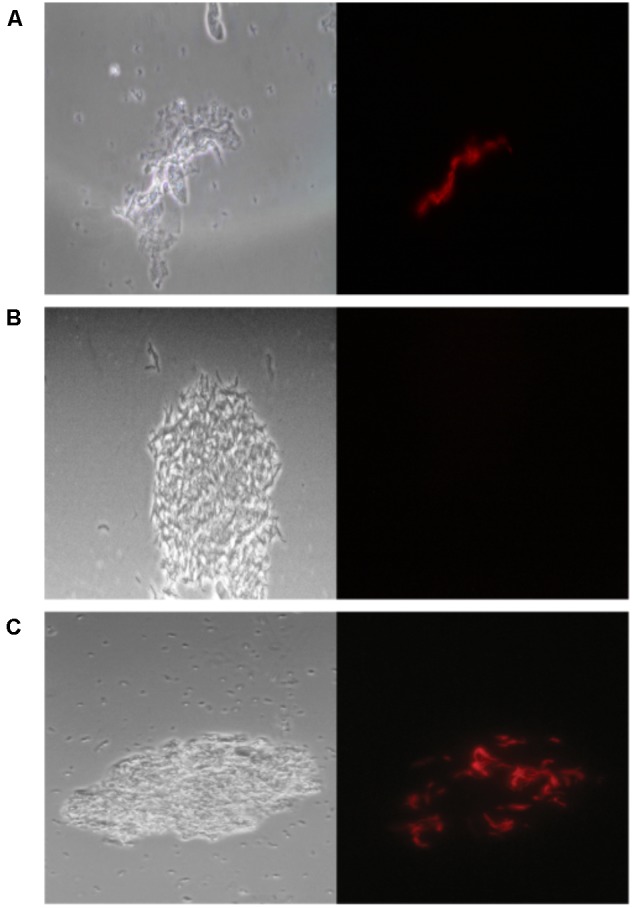
*mCherry_bomb_*ϕ infection of processed sputum samples. **(A)** Fluorescent bacilli can be observed on top of tissue debris, **(B)** not background fluorescence signal is observed in a mock infected control, **(C)** fluorescent bacilli can be easily discriminated in a sample contaminated with coccobacilli. Magnification 100×. Left panel: phase contrast images, right panel: fluorescence micrograph images.

We evaluated the performance of *mCherry_bomb_*ϕ to detect the presence of *Mycobacterium spp.* and determination of RIF resistance in the remnant of 283 sputum samples from presumptive TB patients, treatment-naive, previously treated for TB or under antibiotic treatment for TB. The patient population enrolled in this study is described in **Table 1**. Sputum samples were processed, and part of the pellet was used for routine diagnostic tests and the remnant was used for testing with the fluoromycobacteriophage methodology. The bacteria-containing pellet was recovered in rich media for 48 or 96 h and infected with *mCherry_bomb_*ϕ in the presence or absence of RIF. Samples were fixed with paraformaldehyde, spun down and 100 fields were examined by fluorescence microscopy. The complete protocol is schematized in **Figure 1**. A representative image of a processed sputum sample recovered for 48 h and infected with *mCherry_bomb_*ϕ or mock infected is shown in **Figures 2A,B**. A sample was considered RIF sensitive if fluorescent bacilli were detected in the control sample and fluorescence was lost in the presence of RIF. On the contrary, if fluorescent bacilli were observed in the presence of RIF, the sample was considered resistant to the drug. The sensitivity, specificity, positive and negative predictive values of the bacteriophage method were calculated in comparison to culture in LJ for detection of *Mycobacterium spp.*, or the proportion method for determination of RIF resistance, respectively. The results are summarized in **Tables 2, 3**.

**Table 1:** Demographic and clinical characteristics of enrolled participants.

Characteristics	Description	TB patients^∗^ (% total)
Gender	Male	167 (59)
	Female	114 (40.3)
	Transgender	2 (0.7)
Age	Median (range years)	34
HIV Status	Positive	64 (22.6)
	Negative	135 (47.7)
	Unknown	84 (29.7)
Classification of patients based on treatment	No treatment (Treatment-naive)	40 (14.13)
	Previous TB treatment	56 (19.79)
	In treatment	150 (53)
	Unknown	37 (13.08)
Acid-Fast Bacilli (AFB)	Negative	86 (30.39)
	Positive	197 (69.61)
Culture Status	(−) negative	96 (33.9)
	(+) 1–19 colonies	20 (7.1)
	(++) 20–100 colonies	63 (22.3)
	(+++) >100 colonies	54 (19.1)
	(++++) uncountable colonies	50 (17.7)

*^∗^Presumptive TB patients, under treatment or with any previous treatment.*

**Table 2:** Results and Performance parameters: *mCherry_bomb_*ϕ (Φ) versus culture in LJ media for detection of *Mycobacterium spp.*

(A)					
	**No. of isolates**				
	**Culture (+)**		**Culture (−)**		
**Condition**	**Φ (+)**	**Φ (−)**	**Φ (+)**	**Φ (−)**	**Total**
**48 h ≥ 1**	96	91	0	96	283
**96 h ≥ 1**	172	15	1	95	283

**(B)**					
	**Detection of *Mycobacterium spp.***				
	***mCherry_bomb_*Φ 48 h**	**95% CI**	***mCherry_bomb_*Φ 96 h**	**95% CI**

**All patients**	96/283		172/283	
Sensitivity	51.36	(43.93–58.70)	91.98	(87.12–95.44)
Specificity	100	(96.23–100)	98.96	(94.33–99.97)
PPV	100	(96.23–100)	99.42	(96.82–99.99)
NPV	51.34	(43.93–58.7)	86.36	(78.51–92.16)
**AFB positive**	95/197		170/197	
Sensitivity	52.2	(44.68–59.64)	93.41	(88.77–96.55)
Specificity	100	(78.20–100)	93.33	(68.05–99.83)
PPV	100	(96.19–100)	99.42	(96.78–99.99)
NPV	14.71	(8.47–23.09)	53.85	(33.37–73.41)
**AFB negative**	1/86		2/86	
Sensitivity	20	(0.51–71.64)	40	(52.74–85.34)
Specificity	100	(95.55–100)	100	(95.55–100)
PPV	100	(2.50–100)	100	(15.81–100)
NPV	95.29	(88.39–98.70)	96.43	(89.92–99.26)
**HIV positive**	18/64		36/64	
Sensitivity	48.65	(31.92–65.60)	97.3	(85.84–99.93)
Specificity	100	(87.23–100)	100	(87.23–100)
PPV	100	(81.47–100)	100	(90.26–100)
NPV	58.7	(43.23–73.00)	96.43	(81.65–99.91)
**HIV negative**	49/135		93/135	
Sensitivity	47.12	(37.25–57.15)	89.42	(81.86–94.60)
Specificity	100	(88.78–100)	100	(88.78–100)
PPV	100	(92.75–100)	100	(96.11–100)
NPV	36.05	(25.97–47.12)	73.81	(57.96–86.14)
**Treatment-naive**	2/40		4/40	
Sensitivity	50	(6.75–93.24)	100	(39.76–100)
Specificity	100	(90.26–100)	100	(90.26–100)
PPV	100	(15.81–100)	100	(39.76–100)
NPV	94.74	(82.25–99.36)	100	(90.26–100)
**Previous treatment**	21/56		37/56	
Sensitivity	53.84	(37.18–69.90)	94.87	(82.67–99.37)
Specificity	100	(80.49–100)	100	(80.49–100)
PPV	100	(83.89–100)	100	(90.51–100)
NPV	48.57	(31.38–66.01)	89.47	(66.86–98.70)
**In treatment**	61/150		108/150	
Sensitivity	50.41	(41.17–59.62)	89.25	(82.32–94.15)
Specificity	100	(88.05–100)	96.55	(82.23–99.91)
PPV	100	(94.13–100)	99.08	(94.99–99.98)
NPV	32.58	(23.02–43.34)	68.29	(51.91–81.92)

*(A) Contingency table. (B) The sensitivity, specificity, positive predictive value (PPV), and negative predictive value (NPV) of the fluoromycobacteriophage method were calculated in comparison to culture in LJ.*

**Table 3:** Results and Performance parameters: *mCherry_bomb_*ϕ (Φ) versus proportion method in LJ media for determination of RIF resistance.

(A)					
	**No. of isolates**				
	**Culture (RIF^R^)**		**Culture (RIF^S^)**		
**Condition**	**Φ (RIF^R^)**	**Φ (RIF^S^)**	**Φ (RIF^R^)**	**Φ (RIF^S^)**	**Total**
**48 h ≥ 1**	5	11	0	267	283
**96 h ≥ 1**	16	0	2	265	283

**(B)**					
	**Determination of RIF Resistance**				
	***mCherry_bomb_* Φ 48 h**	**95% CI**	***mCherry_bomb_*Φ 96 h**	**95% CI**

**All patients**	5/283		16/283		
Sensitivity	31.25	(11.02–58.66)	100	(79.41–100)	
Specificity	100	(98.63–100)	100	(98.63–100)	
PPV	100	(47.82–100)	100	(79.41–100)	
NPV	96.04	(93.03–98.01)	100	(98.63–100)	
**AFB positive**	5/197		16/197		
Sensitivity	31.25	(11.02–58.66)	100	(79.41–100)	
Specificity	100	(97.98–100)	100	(97.98–100)	
PPV	100	(47.82–100)	100	(79.41–100)	
NPV	94.27	(89.98–97.11)	100	(97.98–100)	
**AFB negative**	0/86		0/86		
Sensitivity	ND	ND	ND	ND	
Specificity	100	(95.80–100)	100	(95.80–100)	
PPV	ND	ND	ND	ND	
NPV	100	(95.80–100)	100	(95.80–100)	
**HIV positive**	2/64		4/64		
Sensitivity	50	(6.76–93.24)	100	(39.76–100)	
Specificity	100	(94.04–100)	100	(94.04–100)	
PPV	100	(15.81–100)	100	(39.76–100)	
NPV	96.77	(88.83–99.61)	100	(94.04–100)	
**HIV negative**	1/135		9/135		
Sensitivity	11.11	(0.28–48.25)	100	(66.37–100)	
Specificity	100	(97.11–100)	100	(97.11–100)	
PPV	100	(2.50–100)	100	(66.37–100)	
NPV	94.03	(88.58–97.39)	100	(97.11–100)	
**Treatment-naive**	0/40		0/40		
Sensitivity	ND	ND	ND	ND	
Specificity	100	(91.19–100)	100	(91.19–100)	
PPV	ND	ND	ND	ND	
NPV	100	(91.19–100)	100	(91.19–100)	
**Previously treated**	2/56		7/56		
Sensitivity	28.57	(3.66–70.95)	100	(59.03–100)	
Specificity	100	(92.74–100)	100	(92.74–100)	
PPV	100	(15.81–100)	100	(59.04–100)	
NPV	90.74	(79.70–96.92)	100	(92.75–100)	
**In treatment**	3/150				
Sensitivity	37.5	(08.52–75.51)	100	(63.05–100)	
Specificity	100	(97.43–100)	100	(97.43–100)	
PPV	100	(29.24–100)	100	(63.06–100)	
NPV	96.6	(92.24–98.89)	100	(97.44–100)	

*(A) Contingency table. (B) The sensitivity, specificity, positive predictive value (PPV), and negative predictive value (NPV) of the fluoromycobacteriophage method were calculated in comparison to proportion method in LJ.*

For detection of *Mycobacterium spp.*, when the pellet was recovered for 48 h before infection with *mCherry_bomb_*ϕ, 67.84% (192 samples) were concordant between both methods and 32.16% (91 samples) were not detected by *mCherry_bomb_*ϕ but cultured positive in LJ media. An increase in the recovery time to 96 h prior infection improved the detection to 94.35% (267 samples) consistent with both methods. Only 6% of the samples were discordant: 15 tested positive by culture but negative with *mCherry_bomb_*ϕ and 1 was positive by phage and negative by culture (**Table 2A**).

Overall, the sensitivity of *mCherry_bomb_*ϕ for detection of *Mycobacterium spp.* was 51.36% and 91.98% after 48 and 96 h of recovery, respectively, and the specificity was 100% and 98.96% (**Table 2B**). The high specificity was expected because fluoromycobacteriophages can only infect bacteria of the genera *Mycobacterium*. This is a notable advantage of the method because fluorescent *Mycobacterium* bacilli could be easily detected by microscopy even in the presence of other genera contaminants in the sample (**Figure 2C**).

As shown in **Table 2B**, in acid- fast bacilli (AFB) smear-positive patients the sensitivity and specificity were 93.41% and 93.33%, respectively, after recovery for 96 h. The sensitivity in the 86 AFB smear- negative samples was only 40% after 96 h, but it is worth noting that from these samples only five were positive by culture and two of them tested positive with *mCherry_bomb_*ϕ.

Interestingly, for HIV positive patients [that represent 22.61% of the study population and are known to be paucibacillary (Madico et al., 2016; Fan et al., 2017)] the sensitivity and specificity after 96 h reached 97.3% and 100%, respectively.

When the performance of *mCherry_bomb_*ϕ for detection of *Mycobacterium spp.* was calculated for treatment-naive patients or patients previously treated for TB, the values after 96 h of recovery were between 94.87 and 100% for sensitivity and 100% for specificity.

When the same analysis was performed for patients that were currently under antibiotic treatment both parameters drop to 89.25% for sensitivity and 96.55% for specificity after a recovery time of 96 h. From the 15 samples that tested positive by culture and negative by phage (**Table 2A**), 13 corresponded to these groups of patients.

Because fluoromycobacteriophages only infect viable cells that are metabolically active, a likelier explanation for this lower sensitivity is that even after recovery of the sputum sample for 96 h the cells were not in a physiological state to promote *mCherry_bomb_* expression. Alternatively, because we only have access to the remnant of the processed sputum sample, the number of bacteria could be very low in these samples below the level of detection. The reference method used for comparison was culture in LJ medium, which provides all the requirements for optimal growth of mycobacteria, and the time of incubation (30–40 days) allows recovery of bacilli originally present in the sputum sample.

Finally, since we could not discard that Mycobacteria present in those 15 discordant samples were phage resistant, we further prepared liquid cultures from the colonies isolated in LJ media and infected them with *mCherry_bomb_*ϕ. In all samples we visualized bright red fluorescent cells (data not shown) discarding that were phage resistant bacteria.

### Determination of RIF Resistance With *mCherry_bomb_*ϕ in Sputum Samples

Then, we evaluated the performance of *mCherry_bomb_*ϕ to determine susceptibility to RIF. Out of the 283 samples assessed, 16 were identified as RIF^R^ according to the reference method (**Table 3A**).

When we used *mCherry_bomb_*ϕ to determine susceptibility to RIF after a recovery time of 48 h, five samples were identified as RIF^R^ with *mCherry_bomb_*ϕ. In 96.11% of the cases (272 samples) the results for RIF susceptibility were consistent between both methods and in 3.89% (11 samples) were discordant. When recovery time was extended to 96 h, the 16 RIF^R^ samples tested positive with the fluoromycobacteriophage method; the results were congruent for 99.29% of the cases (281 samples) and only 0.71% (2 samples) were discordant (**Table 3A**).

Among the samples determined as RIF^R^, four isolates were identified *a posteriori* as XDR-TB and pre-XDR-TB (see below), illustrating the importance of early detection of resistance to this antibiotic.

Altogether, the sensitivity, specificity and predictive values of *mCherry_bomb_*ϕ to detect rifampicin resistance compared to the proportion method are summarized in **Table 3B**. While the specificity was 100% after recovery for 48 and 96 h, sensitivity highly improved from 31.25%, at 48 h, to 100% when the sample was recovered for 96 h (**Table 3B**).

As can be seen from **Table 3B**, the specificity for detection of rifampicin resistance in all the groups of patients was 100% after recovery for 48 or 96 h.

In AFB smear-positive patients the sensitivity for rifampicin resistance detection reached 100% after recovery for 96 h. The sensitivity in AFB smear- negative samples could not be calculated because none of these samples were RIF^R^.

In HIV positive and negative patients 100% sensitivity was obtained after recovery for 96 h. Once again, sensitivity could not be calculated for treatment-naive patients because none of them carried RIF^R^ strains. For patients under treatment or previously treated for TB, sensitivity highly improved to 100% when cells were recovered for 96 h before infection with *mCherry_bomb_*ϕ.

Overall, it was clear that an extended recovery time was crucial to increase the sensitivity of the fluoromycobacteriophage methodology to determine susceptibility to this first line antibiotic.

### Correlation Between the Number of Bacilli Detected With *mCherry_bomb_*ϕ and the Number of CFU Present in the Sample

**FIGURE 3:**
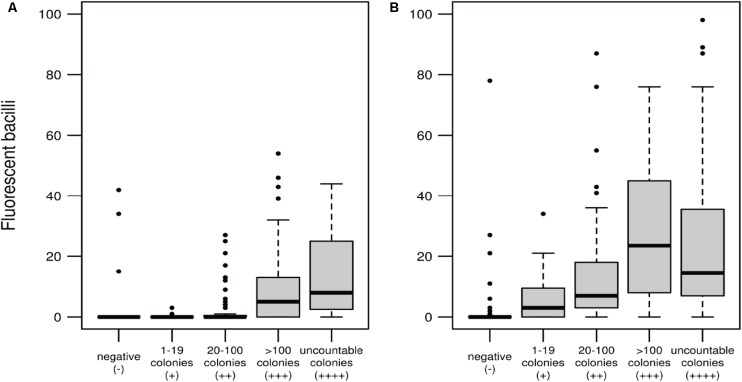
Correlation between the number of fluorescent bacilli detected with *mCherry_bomb_*ϕ and CFU determined by culture in LJ media after recovery of the sputum samples for 48 h **(A)** or 96 h **(B)**. Box and whisker plots: the wide bands represents the medians, the boxes the quartiles Q1 and Q3 (percentiles 25th and 75th, respectively) and the whiskers extend to a maximum of 1.5 the interquartile range, beyond which data are represented with individual points.

One advantage of phenotypic versus genotypic tests is that can discriminate between viable and non-viable cells. This is a key feature for a test intended to monitor patient’s response to antibiotic treatment. Fluoromycobacteriophages can develop a fluorescent signal only in metabolically active cells. As shown in **Figure 3** there is a direct correlation between the number of colonies in LJ and the number of fluorescent bacilli detected by the fluoromycobacteriophage method. When samples were recovered for 48 h before infection, a very low number of fluorescent bacilli (López-Hernández et al., 2016; Sulis et al., 2016) were detected for a colony count between 20 and 100, but this amount increased to 13–27 bacilli per hundred fields when the CFU were more than 100 or uncountable (**Figure 3A**). The number of bacilli detected by *mCherry_bomb_*ϕ notably increased when the recovery time was 96 h and even for those samples with poor growth in LJ media (1–19 CFU) we were able to detect an average of 8 bacilli/100 fields with the phage method (**Figure 3B**) highlighting the good performance of fluoromycobacteriophages in paucibacillary samples when enough time is given for recovery prior to infection.

### Discrimination Between Species of the *M. tuberculosis* Complex and Non-tuberculous Mycobacteria Using PNB

In order to additionally discriminate between MTBC and NTM using *mCherry_bomb_*ϕ, we evaluated the effect of PNB during the assay. As stated, *mCherry_bomb_*ϕ is a derivative of TM4, a broad host range mycobacteriophage (Riska et al., 1997) thus it was not surprising that some of the samples testing positive for *Mycobacterium spp.* with *mCherry_bomb_*ϕ were further identified as containing *M. kansasii*, *M. szulgai*, *M. fortuitum*, *M. abscessus*, and *M. avium.*

**FIGURE 4:**
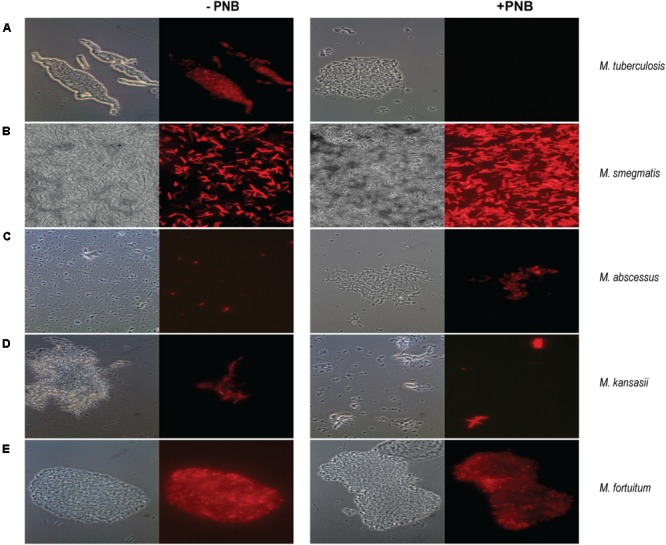
Fluorescence images of *mCherry_bomb_*ϕ infections in presence or absence of *p*-nitrobenzoic acid (PNB) for differentiation of mycobacteria belonging to the MTBC and NTM. Magnification 100×. **(A)** *M. tuberculosis*; **(B)** *M. smegmatis*; **(C)** *M. abscessus*; **(D)** *M. kansasii*; **(E)** *M. fortuitum*. Left panel: phase contrast images, right panel: fluorescence micrograph images.

Cultures of *M. tuberculosis* and atypical mycobacteria were grown until exponential phase and were preincubated with 500 μg/ml of PNB for 24 h prior to infection with *mCherry_bomb_*ϕ. As shown in **Figure 4A**, when *M. tuberculosis* was pretreated with PNB, no fluorescent bacilli were observed. In contrast, infection and expression of *mCherrybomb* was not affected in the NTM strains tested (**Figures 4B–E** and data not shown). These observations suggest that PNB in combination with fluoromycobacteriophages can be used as a simple way to distinguish between MTBC and NTM bacteria present in sputum samples.

### Comparison of Performance of *mCherry_bomb_*ϕ DST of Pre-XDR and XDR Clinical Isolates With Reference Method and WGS

As we previously mentioned, four clinical isolates identified in this study as RIF^R^ using *mCherry_bomb_*ϕ were further reported as XDR or pre-XDR-TB in the lab records.

We evaluated the performance of *mCherry_bomb_*ϕ for extended DST. Resistance to isoniazid, streptomycin, ofloxacin, ethambutol, amikacin, capreomycin, and kanamycin was assessed using liquid cultures of these clinical isolates and results were compared to the proportion method.

We also performed WGS of the four isolates and we searched for mutations in genes reported to confer resistance to the tested antibiotics. The results obtained for the three methodologies are summarized in **Table 4**.

**Table 4:** Phenotypic DST results of pre-XDR and XDR-TB clinical isolates obtained with *mCherry_bomb_*ϕ (Φ) or proportion reference culture method (C).

Strains												
**Antibiotic**	**161109**			**152748**			**16138**			**156324**		
	**Φ**	**C**	**Mutation**	**Φ**	**C**	**Mutation**	**Φ**	**C**	**Mutation**	**Φ**	**C**	**Mutation**
INH	R	R	*katG* S315T	R	R	*kasA* G269S *katG*	R	R	*katG* S315T	R	R	*katG* S315T
						P432L (NR)						
RIF	R	R	*rpoB* D435V	R	R	*rpoB* S450L	R	R	*rpoB* S450L	R	R	*rpoB* S450L
STR	R	R	*rrs* 516C>T	S	S		R	R	*gid*V110fs	R	R	*gid*V110fs
EMB	R	S	*embC* V981L^∗^	R	R	*embB* G406D	R	S	*embB* G406A	R	R	*embB* G406A
						*embC* A774S (NR)			*embC* V981L^∗^			*embC* V981L^∗^
KAN	R	S	No mutation	R	R	No mutation	R	R	*rrs* 1401A>G	R	R	*rrs* 1401A>G
FLQ	R	R	*gyrA* D94H	R	R	*gyrA* A90V	R	R	*gyrA* S95T	R	R	*gyrA* S95T
			*gyrA* S95T			*gyrA* S95T			*gyrA* E21Q (NR)			*gyrA* E21Q (NR)
			*gyrA* E21Q (NR)			*gyrA* E21Q (NR)			*gyrA* G688D (NR)			*gyrA* G688D (NR)
			*gyrA* G688D (NR)			*gyrA* G688D (NR)						

*Putative mutations that could account for the resistance (Mutation) identified after analysis of whole genome sequencing data are indicated. NR, Mutations not reported in bibliography as associated to the indicated antibiotic resistance; S, sensitive; R, resistant. ^∗^Could be S or R depending of the method used for AST.*

For isoniazid and rifampicin we found a perfect correlation between both phenotypic methods and we were able to easily identify mutations that could account for the resistance profile. Genotypic analysis revealed the presence of the *katG* S315T mutation in three isolates and the other presented a *kasA* G269S mutation associated to isoniazid resistance (Lee et al., 1999; Galarza et al., 2014). This strain also had an additional mutation in *katG* (P432L) that has not been previously reported as associated to isoniazid resistance. All RIF^R^ strains had mutations in *rpoB*, three corresponded to the well reported S450L mutation and the other was a change from aspartic acid to valine in position 435 (D435V) (Eldholm et al., 2015; Datta et al., 2016).

For streptomycin, we found a good correlation between the fluoromycobacteriophage and the proportion method and we were able to identify the gene variants that could be responsible for the observed phenotype. One of the strains presented a mutation in the 16S rRNA gene, *rrs* 516C>T (Dobner et al., 1997). The other two strains had a frameshift in *gidB* that is a well-established determinant of streptomycin resistance (Okamoto et al., 2007). Moreover, the *gidB* V110 frameshift was previously found in 90% of analyzed isolates of the M strain that caused an outbreak in Argentina in the early 1990s (Eldholm et al., 2015). Streptomycin was introduced in anti TB regimens in Argentina in 1946 (de Kantor and Barrera, 2007) so it is important to include this antibiotic during DST.

Results for ethambutol were more discrepant. Two strains were sensitive by the reference method and resistant by *mCherry_bomb_*ϕ. The strain 161109 had an *embC* V981L mutation that could render the strain sensitive or resistant depending of the method use for DST (Sreevatsan et al., 1997). Strain 16138 also had an *embB* G406A mutation in addition to that but tested sensitive to ethambutol with the proportion method. Antibiotic susceptibility testing was repeated in this strain, with the same outcome. This result was unexpected because strain 156324 presented the same gene variants for *embB* and *embC* and was resistant to ethambutol by phage and the proportion method. It has been previously reported that testing of resistance to ethambutol can be inconsistent and can lead to ambiguous results (Madison et al., 2002).

For kanamycin and the other two injectable antibiotics tested, amikacin and capreomycin (data not shown), we got similar results for both phenotypic methods with the exception of isolate 161109 that tested sensitive for kanamycin by the reference method and resistant by the phage method. The sample from this patient was also tested using MGIT and some bacterial growth was observed at day 12 in the presence of kanamycin. This could be indicative of the presence of a mixed population with the Kan^R^ population present in a very low proportion. It is important to notice that fluoromycobacteriophages can detect individual cells being particularly useful for detection of partially resistant cultures even if resistant cells are underrepresented in the sample. For two of the resistant isolates we identified the *rrs*1401A>G mutation associated to kanamycin resistance but for strain 152748 and also for isolate 161109 we were not able to find any mutations that could be associated to this phenotype.

All the strains resulted resistant to at least one fluoroquinolone, either with *mCherry_bomb_*ϕ or the reference method. In all the strains we identified mutations in *gyrA* previously reported as associated to fluoroquinolone resistance. Additionally, all four isolates presented the following mutations: *gyrA* E21Q and *gyrA* G688D but we were not able to find any reports describing these mutations as responsible for resistance to fluoroquinolones.

**FIGURE 5:**
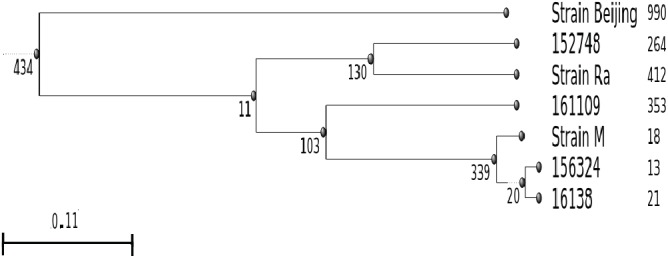
Dendrogram based on SNPs between the pre-XDR and XDR strains isolated in this study (16138, 156324, 161109, and 152748) and clinical isolates of two of the largest outbreaks in Argentina (M, Ra). Number of SNPs were calculated using the H37Rv strain as reference, the Beijing strain was included as out-group. Figures next to each strain indicate number of unique SNPs.

Finally, we performed a phylogenetic analysis in an attempt to trace these four isolates. For this analysis we constructed a dendrogram based on SNPs between these strains and clinical isolates of two of the largest TB outbreaks in Argentina with available reads at NCBI, ERR760768, and ERR776666, reported as M (of Haarlem family) and Ra (of LAM- Latin American Mediterranean family), respectively (Eldholm et al., 2015; Bigi et al., 2017) (**Figure 5**). The analysis of the dendrogram shows that 156324 and 16138 cluster in the same group as the M strain. However, strain 152748 was closer to Ra than other analyzed strains. Strain 161109 did not cluster with any of the analyzed strains.

## Discussion

Several phage-based methods have been proposed for TB diagnosis over time but there are few studies validating their use in sputum samples (Banaiee et al., 2001; Albert et al., 2004, 2007; Kiraz et al., 2007; Mole et al., 2007; Zhu et al., 2011; Hemvani et al., 2012; Jain et al., 2012; O’Donnell et al., 2015).

When we evaluated the performance of *mCherry_bomb_*ϕ compared to the reference method to detect *Mycobacterium spp.* in sputum, the sensitivity and specificity after recovering the sample for 96 h reached values of 91.98 and 98.96%, respectively.

The most recent work using reporter phages carrying fluorescent proteins in combination with flow cytometry for detection in sputum, informed a mean time for results of 7.5 days (range 5–9 days) and a sensitivity and specificity for *M. tuberculosis* of 95.90% and 83.33%, respectively (O’Donnell et al., 2015).

In this work, the high sensitivity and specificity values for *mCherry_bomb_*ϕ were achieved using only part of the processed sputum sample remaining after its use for routine practices in the clinical lab, and these parameters are likely to be improved with access to a standardized sample. However, an advantage of the study design is that evaluation of fluoromycobacteriophage technology did not require changes to the routine processing of samples in the clinical laboratory. Furthermore, fluorescence microscopy is available in many clinical laboratories, and fixation of cells with paraformaldehyde reduces biohazard concerns.

During experimental design, recovery time before infection was a key parameter as demonstrated by the approximately 40% increment in sensitivity at 96 h versus 48 h. Even though our study suggests that optimal results are obtained after recovering for 96 h, in the practice results for 50% of the samples can be obtained after just 48 h of recovery (3 days from sputum collection). This time point should be tested if enough sample is provided for analysis.

With this experimental set up, in just 3 days from sputum collection, 49% (18 of 37) of the samples positive by culture from HIV patients tested positive with the fluoromycobacteriophage method. After 5 days, 97% (36 of 37) of the samples were detected, reflecting the usefulness of this technique for TB diagnosis in paucibacillary samples.

For determination of rifampicin resistance, the sensitivity and specificity of *mCherry_bomb_*ϕ were both 100%, when the sample was recovered for 96 h prior to infection. The marked difference in sensitivity, from 31.25 to 100% when the sample was recovered for 96 h instead of 48 h, may reflect either a heterogeneous distribution of bacilli in the sample or the presence of mixed populations of rifampicin sensitive and resistant bacteria additionally to the observation that an extended recovery time can improve the metabolic state and number of viable bacilli in the sample as explained above.

The high specificity obtained with *mCherry_bomb_*ϕ to detect *Mycobacterium spp.* was not unexpected because bacteriophages have a limited host range, mostly restricted to bacteria belonging to the same genera. The high sensitivity of *mCherry_bomb_*ϕ for detection of mycobacteria and rifampicin resistance was evidenced by the fact that all the strains in this study that were RIF^R^ by culture, were identified by *mCherry_bomb_*ϕ. In general terms is crucial that a diagnostic test shows a high sensitivity, capturing all the active TB patients to implement the proper treatment and dissemination of resistant strains, but also a high specificity, as demonstrated for *mCherry_bomb_*ϕ, to avoid uninfected people being exposed to unnecessary and long treatments with several adverse effects (Salzer et al., 2016; Dobbs and Webb, 2017).

Because phage *mCherry_bomb_*ϕ infects both MTBC and NTM, and the treatment of such infections is very different (Salzer et al., 2016; Wassilew et al., 2016), in a standardized sputum sample one extra aliquot could be directly treated with PNB (Shakoor et al., 2010) for early distinction between these mycobacteria. We note that fluoromycobacteriophages based on phage DS6A – which only form plaques on MTBC strains – also give reporter signal from some NTM strains (Mayer et al., 2016), and thus a pre-infection treatment with PNB remains a reasonable option for strain discrimination.

Since rapid genotypic tests cannot discriminate between viable and non-viable cells, the good correlation found between the number of viable bacteria recovered from sputum and the number of fluorescent bacilli detected with *mCherry_bomb_*ϕ suggests that this methodology could be a promising tool for a rapid follow up of patient’s response to antibiotic treatment.

During this study, using *mCherry_bomb_*ϕ we identified all samples carrying RIF^R^ strains after 5 days from sputum collection. Four of those strains were further classified by culture as pre-XDR and XDR-TB reflecting the importance of early detection of this marker. When susceptibility to other antibiotics was tested with *mCherry_bomb_*ϕ in these four isolates, a good correlation was observed with the results from the reference proportion method. It is worth noticing that if enough sample is provided for analysis, susceptibility to any antibiotic could be tested directly in the recovered sputum sample based on the incidence of antibiotic resistance in the tested population.

After WGS of the four pre-XDR and XDR-TB strains, in most cases we were able to identify mutations that could explain the observed phenotype. But for one strain that tested kanamycin resistant with both methods (152748), we were not able to identify genes that could account for that profile despite detailed analysis of the sequencing data. This result highlights the discrepancies that can be found between genotypic and phenotypic tests (Ocheretina et al., 2014; Ahmad et al., 2016; Gurbanova et al., 2017). Antibiotic resistance cannot always be correlated with the presence of mutations in expected genes (Louw et al., 2009), which is the case of efflux-mediated resistance (da Silva et al., 2011; Machado et al., 2018) and also, it has been shown, for example, that mycobacterial mistranslation has a role in tolerance to the antibiotic rifampicin due to the generation of gain of function protein variants where the *rpoB* gene is unaffected (Javid et al., 2014). These observations reflect the importance of rapid methods that evaluate phenotypic resistance in clinical isolates.

The estimated cost for a *mCherry_bomb_*ϕ based diagnostic test is about USD 2 for the four determinations (48/96 h ±RIF). Since this is a phenotypic test, susceptibility to any antibiotic can be evaluated, including those with mechanisms of antibiotic resistance that cannot be identified using a molecular beacon-based approach (Rubin, 2018) or when genetic determinants for antibiotic resistance are not known.

The demonstrated attributes of *mCherry_bomb_*ϕ could facilitate making this methodology an easy, inexpensive and rapid complementary phenotypic assay for TB diagnosis and DST to be implemented in moderate risk TB laboratories (World Health Organization, 2012).

## Author Contributions

LR, CL, EU, FP, MM, and SP contributed to concept and design, acquisition of data, data analysis and interpretation, and drafting the manuscript. ES, DDP, AT, and SN contributed to sequencing data analysis and interpretation. GH contributed to data analysis and interpretation, and drafting and revising of the manuscript. MP contributed to concept and design, data analysis and interpretation, drafting and revising, and approval of the manuscript.

## Conflict of Interest Statement

The authors declare that the research was conducted in the absence of any commercial or financial relationships that could be construed as a potential conflict of interest.
